# Genetic insights into the gut microbiota and risk of psoriasis: a bidirectional mendelian randomization study

**DOI:** 10.3389/fmicb.2024.1434521

**Published:** 2024-08-05

**Authors:** Minyu Qian, Jianxin Shi, Zhuoya Zhang, Dezhao Bi, Cheng Tan

**Affiliations:** Department of Dermatology, Affiliated Hospital of Nanjing University of Chinese Medicine, Jiangsu Province Hospital of Chinese Medicine, Nanjing, China

**Keywords:** bidirectional, causal association, gut microbiota, Mendelian randomization study, psoriasis

## Abstract

**Background:**

Growing evidence indicates a potential association between the gut microbiome and psoriasis. Nevertheless, the precise nature of these associations and whether they constitute causal relationships remain unclear.

**Methods:**

A rigorous bidirectional two-sample Mendelian randomization study was undertaken to establish a putative causal link between gut microbiota and psoriasis. We drew upon publicly available datasets containing summary statistics from GWAS to accomplish this. Utilizing various analytical techniques, including inverse variance weighting, MR-Egger, weighted median, weighted model, and MR-PRESSO, we sought to validate the putative causal association between gut microbiota and psoriasis. A reverse Mendelian randomization analysis was conducted to further investigate the relationship.

**Results:**

After conducting a forward Mendelian randomization analysis, a causal relationship was established between 19 gut microbiota and psoriasis. Furthermore, the reverse MR study revealed causality between psoriasis and 13 gut microbiota. Notably, no substantial heterogeneity of instrumental variables or horizontal pleiotropy was observed.

**Conclusion:**

This research suggests a potential genetic association and causal nexus between gut microorganisms and psoriasis, indicating potential implications for the clinical management and therapy of psoriasis. Additional observational studies with a larger population sample size and animal model experiments are imperative to fully elucidate this association’s underlying mechanisms.

## 1 Introduction

Psoriasis is a chronic and immune-mediated inflammatory skin disease characterized by intermittent flare-ups of red, scaly patches (Boehncke and Schön, 2015). Psoriasis affects approximately 2% to 4% of the global population, varying prevalence across different geographical regions and ethnic groups (Parisi et al., 2013). The precise reasons behind psoriasis remain enigmatic, and it is widely accepted that a combination of genetic predisposition and environmental risk factors plays a significant role in its etiology. Psoriatic patients are exposed to an increased risk of developing a range of chronic and severe health conditions, including colorectal cancer, metabolic syndrome, obesity, nonalcoholic fatty liver disease, Crohn’s disease, lymphoma, and cardiovascular diseases (Gulliver, 2008). Due to its recurrent nature, Psoriasis remains incurable, thereby exerting considerable economic pressure on patients and healthcare systems.

The gut microbiota serves as a crucial regulator of immune responses (Xiao et al., 2021). The gut-skin axis, referring to the bidirectional relationship between the gut microbiome and skin health, has emerged as a noteworthy field of investigation. Microbiota dysregulation has been documented across diverse inflammatory skin disorders (Thye et al., 2022). Recent studies have shed light on the intricate relationship between the gut microbiome and psoriasis, suggesting that the gut-skin axis plays a crucial role in the disease’s pathogenesis (Shapiro et al., 2007). Microbiota dysbiosis might trigger abnormal immune responses in psoriasis, suggesting that disturbances in gut flora could act as triggers or causes for recurrent psoriasis episodes (Zhang et al., 2021). Multiple studies have consistently demonstrated disparities in the gut microbial composition between psoriatic patients and those who are healthy. Among the significant observations, there are specific alterations in the abundance of various bacterial genera and phyla. Expressly, a review article examined the relationship between the abundance of *Firmicutes* (synonym *Bacillota*) and *Bacteroidetes* (synonym *Bacteroidota*) phyla in psoriatic patients compared to a healthy control group. In all cases of psoriasis, the *Firmicutes*/*Bacteroidetes* (F/B) ratio was elevated compared to the control group (Polak et al., 2021). It was discovered that dysregulation of the gut microbiota, caused by vancomycin and polymyxin, worsened psoriasis in a mouse model induced with imiquimod (Zanvit et al., 2015). Treatments for psoriasis seem to impact the gut microbiome’s composition. Oral administration of *Lactobacillus pentosus GMNL-77* significantly reduces psoriasis-like skin lesions in mice (Chen et al., 2017).

Despite these observations, the causal links between gut microbiota alterations and psoriasis remain enigmatic. Potential confounding factors could potentially obfuscate the conclusions. Statistical approaches leveraging genome-wide association studies (GWAS) have recently emerged to assess the association and causality between traits. Mendelian randomization (MR) presents a promising avenue to tackle these complexities (Davey Smith and Ebrahim, 2003). This analytical approach uses genetic variants associated with modifiable risk factors as instrumental variables to infer causal relationships with disease outcomes. By leveraging the random assortment of genes from parents to offspring at conception, MR mimics the randomization process of a controlled trial, thereby minimizing confounding and providing a more objective assessment of causality (Hemani et al., 2018). MR analysis further enhances the feasibility and efficiency of exploring the gut microbiota’s causal role in psoriasis.

The objective of this study is to meticulously evaluate the genetic interplay and causal connections between the gut microbiome and psoriasis. This endeavor aims to establish a robust foundation for future clinical implementations and advance the dermatological research domain.

## 2 Materials and methods

### 2.1 Study design

**FIGURE 1:**
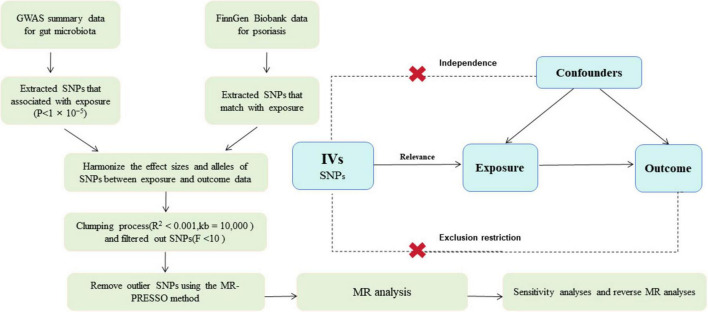
Overview of present MR analyses and assumptions. GWAS, genome wide association study; SNP, single nucleotide polymorphisms; MR, Mendelian randomization.

We conducted two-sample Mendelian randomization (2SMR) analysis to investigate the associations between gut microbiota and psoriasis. The comprehensive details of this study are illustrated in Figure 1. For the 2SMR analysis to yield reliable results, it must adhere to three fundamental assumptions: strong correlation, independence, and exclusion restriction assumptions (Davey Smith and Hemani, 2014). The instrumental variables (IVs) utilized in the 2SMR analysis are also required to meet these criteria, ensuring their validity. To mitigate the issue of reverse causality, which could compromise causal inference, reverse 2SMR analysis was employed. The study design and reporting conformed to STROBE-MR (Supplementary Table 1; Skrivankova et al., 2021). All statistical analyses were performed using R (version 4.3.2).

### 2.2 Data sources for psoriasis

GWAS data for psoriasis were acquired from the latest data version by the FinnGen consortium^1^ in December 2023. Its total sample size was 412,181(230,310 females and 181,871 males), with an analyzed total of 21,311,942 variants and 2,408 available disease endpoints. The GWAS statistics for psoriasis included 10,312 cases and 397,564 controls from a prospective cohort study involving the European population. The diagnosis of psoriasis was firmly established following the ICD-10 standards. The FinnGen study received ethical approval, and all participants provided their informed consent before involvement. The dataset is available at all times to support further research and analysis.

### 2.3 Data sources for gut microbiome

Complete summary statistics of gut microbial taxa with genome-wide significant hits are publicly available in the NHGRI-EBI GWAS Catalog^2^ from accession GCST90032172 to GCST90032644. Genetic variants associated with the gut microbiome were obtained from genome-wide association tests applied to 7,979,834 human genetic variants from the 5,959 individuals enrolled in the FINRISK 2002 cohort. This cohort includes people aged 24–74 years (mean = 49.61) from six regions of Finland, with a sex distribution of 55.1% women and 44.9% men (Table 1). Genome-wide association analysis of gut microbial taxa identifies 567 independent Single nucleotide polymorphisms (SNP)-taxon associations (Qin et al., 2022).

**TABLE 1:** Baseline characteristics of data sources for gut microbiome analysis.

Characteristic	Value
Study name	FINRSK 2002
Total participants	8798
Participation rate (%)	65.5
Age (years)	24–74 (mean = 49.61)
Sex (%)	Women 55.1, Men 44.9
Geographical areas	Six areas of Finland
Sampling method	Stratified by sex, region, and 10-year age group with 250 participants per stratum

### 2.4 Instrumental variable selection

The selection of eligible genetic IVs was conducted with stringent criteria to ensure reliable causality between the gut microbiota and psoriasis. (1) Considering that the number of available IVs at *p* < 5 × 10^–8^ was relatively limited, a comprehensive threshold of *p* < 1 × 10^–5^ was set to obtain a relatively large number of IVs. (2) A clumping process was implemented (R^2^ < 0.001, window size = 10,000 kb) to remove variants that were in tight linkage disequilibrium to guarantee the independence of IVs. (3) Palindromic SNPs and those with ambiguous allele effects were removed to prevent misinterpretation of the genetic influence on the exposure. (4) The strength of the selected IVs was critically evaluated using F-statistics, excluding any inferior SNPs with a minor allele frequency (F-statistics < 10). F = R^2^× (N–1–K) (1–R^2^) × K.

## 3 Statistical analysis

### 3.1 MR analysis

A rigorous and comprehensive analytical approach was employed, encompassing five methods to assess the causal association between gut microbiota and psoriasis. The primary method utilized was the Inverse Variance Weighted (IVW) approach. Depending on the heterogeneity of the data, we either employed the IVW random-effects model (IVW-RE) for heterogeneous datasets or the IVW fixed-effects model (IVW-FE) for homogenous datasets. MR-Egger provides a complementary analysis for IVW (Bowden et al., 2015). The MR-Egger intercept test was specifically used to evaluate the existence of horizontal pleiotropy, with *p* > 0.05 indicating its absence. Weighted Median allowed for a reliable causal inference even when up to 50% of the instrumental variables might be invalid, adding another layer of robustness to our analysis (Burgess and Thompson, 2013). Simple Mode and Weighted Mode methods offer robust causal estimates under varying assumptions about the validity of the instrumental variables. The Simple Mode method gives the most common causal estimate among the selected SNPs, while the Weighted Mode provides a weighted average, prioritizing more robust instruments. When the results analyzed by the 2SMR study were statistically significant (*p* < 0.05), it was suggested that there may be a causality between the gut microbiota and inflammatory skin diseases.

### 3.2 Sensitivity analysis

In our study, both the IVW and MR-Egger methods utilized Cochran’s Q-test to evaluate heterogeneity among IVs. A *p*-value < 0.05 indicated significant heterogeneity across IVs. We conducted an iterative leave-one-out analysis, omitting each SNP in turn to determine the impact of individual SNPs. We also probed for horizontal pleiotropy through the MR-Egger intercept, considering it negligible for our causal conclusions if its *p*-value > 0.05. Furthermore, we applied MR-PRESSO global test to discern pleiotropy, with a *p*-value > 0.05 suggesting its absence, and eliminated outliers based on this (Xiang et al., 2021).

### 3.3 Reverse mendelian randomization analysis

To better understand the causal association between gut microbiota and diseases, we employed reverse MR analysis, treating psoriasis as the exposure variable and gut microbiota as the outcome. The analysis was conducted using identical methods and settings as those employed in the forward analysis.

## 4 Results

### 4.1 Results of the causal effect of gut microbiota on psoriasis

**FIGURE 2:**
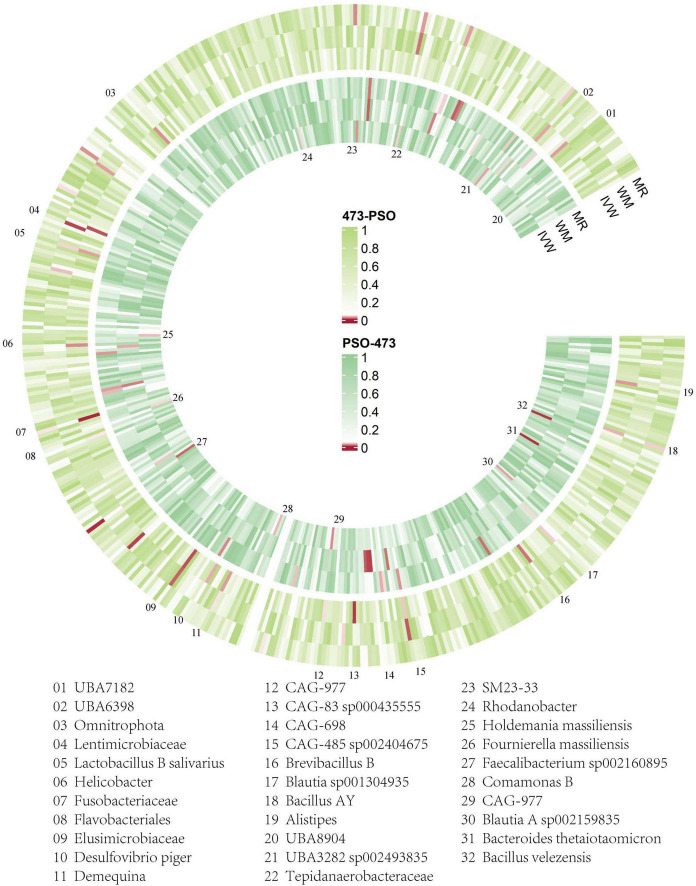
Overview of the causal role of gut microbiota and psoriasis in MR analysis (outer section) and reverse MR analysis (inner section). The red color indicates statistical significance (*p* < 1 × 10^–5^). ME, MR-Egger method; WM, weighted median method; IVW, inverse variance weighted method.

We conducted the formal MR analysis to explore the association between 473 gut bacterial taxa and psoriasis. The analysis revealed that all SNPs considered had F-values exceeding 10, indicating a significant correlation with the gut microbiota. Harmonization of the data led to the identification of SNPs that were subsequently employed as IVs in the MR analysis. We introduced a circular heat map to visually illustrate the strength of the IVs and enhance data comprehension (Figure 2). The complete list of IVs used in the identified causal associations is provided in Supplementary Table 2. We identified 19 bacterial taxa associated with psoriasis. These bacterial taxa encompassed one phylum (with 20 SNPs), one order (with 25 SNPs), five families (with 128 SNPs), seven genera (with 136 SNPs), and five species (with 102 SNPs). Notably, no causal association was observed at the class level.

**FIGURE 3:**
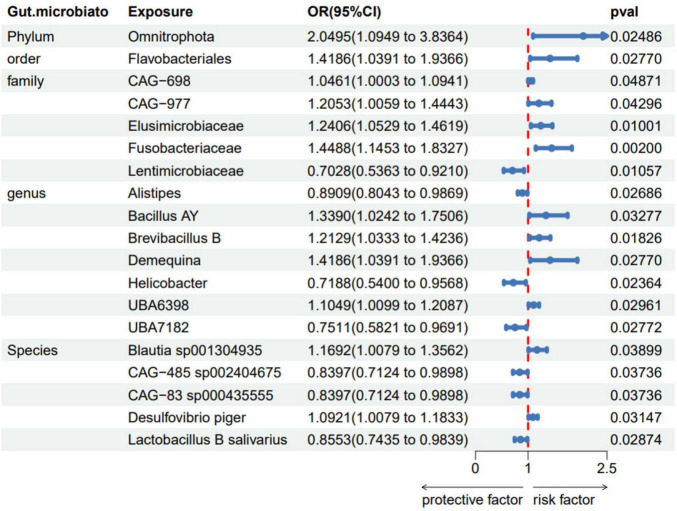
Forest plots of gut microbiota taxa associated with psoriasis identified by IVW method. SNP, single-nucleotide polymorphism; OR, odds ratio; CI, confidence Interval.

The fixed-effects Inverse Variance Weighted (IVW-FE) analysis revealed that specific microbiota act as protective factors, including family *Lentimicrobiaceae* (OR = 0.703, 95%CI: 0.536–0.921, *p* = 0.011), genus *Alistipes* (OR = 0.891, 95% CI: 0.804–0.987, *p* = 0.027), genus *Helicobacter* (OR = 0.719, 95% CI: 0.540–0.957, *p* = 0.024), genus *UBA7182* (OR = 0.751, 95% CI: 0.582–0.969, *p* = 0.028), species *Lactobacillus B salivarius* (OR = 0.855, 95% CI: 0.743–0.984, *p* = 0.029), species *CAG-83 sp000435555* (OR = 0.840, 95% CI: 0.712–0.990, *p* = 0.037), species *CAG-485 sp002404675* (OR = 0.855, 95% CI: 0.743–0.984, *p* = 0.029). Conversely, phylum *Omnitrophota* (OR = 2.050, 95% CI: 1.095–3.836, *p* = 0.025), order *Flavobacteriales* (OR = 1.419, 95% CI: 1.039–1.937, *p* = 0.028), family *Elusimicrobiaceae* (OR = 1.241, 95% CI: 1.053–1.462, *p* = 0.010), family *Fusobacteriaceae* (OR = 1.449, 95% CI: 1.145–1.833, *p* = 0.002), family *CAG-698* (OR = 1.046, 95% CI: 1.000–1.094, *p* = 0.049), family *CAG-977* (OR = 1.205, 95% CI: 1.006–1.444, *p* = 0.043), and several genera and species including genus *Bacillus AY* (OR = 1.339, 95% CI: 1.024–1.751, *p* = 0.033), genus *Brevibacillus B* (OR = 1.213, 95% CI: 1.033–1.424, *p* = 0.018), genus *Demequina* (OR = 1.419, 95% CI: 1.039–1.937, *p* = 0.028), genus *UBA6398* (OR = 1.105, 95% CI: 1.010–1.209, *p* = 0.030), species *Blautia sp001304935* (OR = 1.169, 95% CI: 1.008–1.356, *p* = 0.039), and species *Desulfovibrio piger* (OR = 1.092, 95% CI: 1.008–1.183, *p* = 0.031) were associated with an increased risk of psoriasis (Figure 3).

Detailed results are available in Supplementary Table 3. Each association was supported by statistical significance in *p*-values (*p* < 0.05) and narrow confidence intervals (95%CI), underscoring links between these specific gut microbiota and the occurrence of psoriasis.

Sensitivity analysis signified no discernible heterogeneity among the selected SNPs (*p* > 0.05) in Cochran’s Q Test. MR-Egger analysis further confirmed the absence of horizontal pleiotropy (Table 2). Moreover, MR-PRESSO test indicated that no single SNP disproportionately influenced the analysis. Detailed results are available in Supplementary Table 4. Scatter plots and the “leave-one-out” approach proved the results’ stability. Forest plots for the causal effects of gut microbes on psoriasis are listed in Supplementary Figures 1–4. Funnel plots for MR causal effects of gut microbes on psoriasis are listed in Supplementary Figures 5–8. Supplementary Figures 9–12 show the scatter plots, and Supplementary Figures 13–16 shows the “leave-one-out” analyses. These findings underscore a reliable association between specific gut microbiome components and psoriasis.

**TABLE 2:** Sensitivity analysis of 19 taxa associated with psoriasis.

Exposure	Outcome	SNP	MR-egger intercept		Cochrane’s Q IVW		Cochrane’s Qegger		PRESSO global test *P*
			Intercept	*P*-value	*Q* value	*P*-value	*Q*-value	*P*-value	
*Omnitrophota*	Psoriasis	20	−0.00868719	0.42211304	15.94386063	0.66102035	15.26900845	0.64342068	0.662
*Flavobacteriales*	Psoriasis	17	−0.01189862	0.25097824	25.70647630	0.36822461	24.24456869	0.39037086	0.365
*CAG-698*	Psoriasis	25	−0.00072673	0.90334822	21.58270173	0.60415831	21.56762691	0.54646529	0.802
*CAG-977*	Psoriasis	25	0.00927290	0.39246967	22.12854170	0.57159471	21.36897075	0.55856592	0.614
*Elusimicrobiaceae*	Psoriasis	34	−0.00557137	0.56088179	31.88342662	0.52257313	31.53807682	0.48981437	0.521
*Fusobacteriaceae*	Psoriasis	24	−0.00712070	0.51099132	17.92779570	0.76139401	17.48137940	0.73625641	0.777
*Lentimicrobiaceae*	Psoriasis	19	−0.01649743	0.26690370	16.90420530	0.52970032	15.58650930	0.55331314	0.559
*Alistipes*	Psoriasis	18	−0.02077435	0.10461234	19.11001297	0.32225420	16.12638052	0.44417568	0.337
*Bacillus AY*	Psoriasis	20	−0.01511030	0.17663337	17.38352482	0.56390220	15.40554679	0.63395849	0.599
*Brevibacillus B*	Psoriasis	19	−0.00003228	0.99793284	12.13154805	0.84037192	12.13154114	0.79210722	0.84
*Demequina*	Psoriasis	17	0.00291054	0.81017387	12.50442894	0.70858770	12.44465981	0.64510765	0.703
*Helicobacter*	Psoriasis	18	−0.00046688	0.97216158	16.11215535	0.51589845	16.11089013	0.44524861	0.543
*UBA6398*	Psoriasis	23	−0.00454876	0.73124473	20.18364146	0.57155366	20.06248142	0.51730673	0.603
*UBA7182*	Psoriasis	19	0.01657450	0.21328347	24.57710343	0.13701512	22.37628674	0.17067761	0.158
*Blautiasp001304935*	Psoriasis	16	0.02617420	0.06402787	11.26451843	0.73362763	7.22167608	0.92582804	0.757
*CAG-485sp002404675*	Psoriasis	18	−0.00308902	0.84082205	11.09398007	0.85163692	11.05230903	0.80624507	0.86
*CAG-83sp000435555*	Psoriasis	18	−0.03092123	0.22675220	14.06116554	0.36955054	12.38567401	0.41522361	0.394
*Desulfovibrio piger*	Psoriasis	24	−0.00129882	0.92094856	31.70462191	0.10644391	31.69010644	0.08283499	0.106

SNP, single-nucleotide polymorphism; IVW, inverse-variance weighted; MR, Mendelian randomization.

### 4.2 Reverse MR analysis

A reverse MR analysis was conducted, treating significant gut microbiota as the dependent variable and psoriasis as the independent variable. Supplementary Table 5 provides the IVs used. According to the IVW method, family *CAG-977* (OR = 0.986, 95% CI:0.975–0.998, *p* = 0.023), family *Tepidanaerobacteraceae* (OR = 0.986, 95% CI: 0.973–0.999, *p* = 0.040), genus *Comamonas B* (OR = 0.990, 95% CI: 0.981–0.999, *p* = 0.034), and species *Bacteroides thetaiotaomicron* (OR = 0.959, 95% CI: 0.931–0.987, *p* = 0.004) had a positive causal effect on the gut microbiota. We also found that psoriasis was linked to a decreased relative abundance of order SM23-33 (OR = 1.010, 95% CI: 1.001–1.019, *p* = 0.025), genus *UBA8904* (OR = 1.009, 95% CI: 1.000–1.018, *p* = 0.047), genus *Rhodanobacter* (OR = 1.008, 95% CI: 1.000–1.016, *p* = 0.042), species *Bacillus velezensis* (OR = 1.016, 95% CI: 1.005–1.027, *p* = 0.005), species *Blautia A sp002159835* (OR = 1.019, 95% CI: 1.002–1.036, *p* = 0.029), species *UBA3282 sp002493835* (OR = 1.013, 95% CI: 1.001–1.024, *p* = 0.029), species *Faecalibacterium sp002160895* (OR = 1.013, 95% CI: 1.003–1.024, *p* = 0.015), species *Fournierella massiliensis* (OR = 1.012, 95% CI: 1.000–1.023, *p* = 0.041), and species *Holdemania massiliensis* (OR = 1.026, 95% CI: 1.002–1.051, *p* = 0.035). The results of reverse MR analysis are presented in Supplementary Table 6. None of the above analyses revealed heterogeneity or pleiotropy (Supplementary Tables 6, 7).

## 5 Discussion

We employed the most current and comprehensive Genome-Wide Association Studies datasets available, encompassing 473 gut microbiota taxa classified down to the species level, providing more extensive analysis results than previous studies. A Mendelian Randomization study investigated the association between gut microbiota and psoriasis. The research identified 19 bacterial taxa that could potentially exert a causal influence on psoriasis. Additionally, a reverse MR study revealed a putative causal relationship between psoriasis vulgaris and 13 taxa of gut microbiota. These findings provide valuable insights into the pathophysiology of psoriasis and establish a theoretical basis for future therapeutic interventions.

The balance and composition of the gut microbiome play a pivotal role in maintaining overall health. Recent scientific investigations have increasingly delved into the mutual influence between skin homeostasis and gastrointestinal well-being. The majority of these studies reveal notable disparities in the gut microbiome of psoriasis patients versus those of healthy individuals (Polak et al., 2021). Sonomoto et al. (2023) found individuals with psoriasis exhibit increased levels of *Prevotella* and reduced levels of *Lachnospira* and *Akkermansia muciniphila*. Additionally, these patients typically show lower overall biological diversity compared to healthy controls (Sonomoto et al., 2023). However, the variation trends in the abundances of *Firmicutes*, *Actinobacteria*, and *Proteobacteria* across various studies were highly controversial (Chen et al., 2020). It is hypothesized that a lower F/B ratio among type 2 enterotype psoriasis patients may be associated with an increased risk of bacterial translocation. Such bacterial translocation can potentially lead to pro-inflammatory reactions and subsequent skin inflammation (Codoñer et al., 2018).

Different studies have reported varying trends in the abundance of intestinal flora. Similarly, our study both aligns with and diverges from previous findings, presenting some consistent results as well as new insights.

We discovered that the family *Lentimicrobiaceae* has protective effects on psoriasis. Additionally, Two genera of gut microbiota, *Alistipes*, and *Helicobacter*, along with specific species have been associated with a reduced risk of psoriasis. Our analysis suggests that *Alistipes* and *Lactobacillus B* salivarius serve as protective factors for psoriasis, in line with its noted reduction in relative abundance in prior studies (Scher et al., 2015; Hidalgo-Cantabrana et al., 2019). *Alistipes* produces SCFAs, which are known to modulate the immune system (Kaur et al., 2017). Gut microbiota produces beneficial metabolites like butyrate, which reduces oxidative stress, provides energy for colonocytes, and activates regulatory T cells (Treg) to promote anti-inflammatory actions and enhance immune tolerance throughout the body. Research indicates that psoriasis is often associated with a reduced presence of bacteria that produce short-chain fatty acids (SCFAs) (Scher et al., 2015). These SCFAs play crucial roles in modulating the host’s immune system by suppressing pro-inflammatory cytokines and promoting Treg differentiation (Xiao et al., 2023). Recent research has revealed that an imbalance, or dysbiosis, of *Alistipes* can have either protective or detrimental effects. *Alistipes* may contribute to mitigating conditions such as colitis, autism spectrum disorder, and fibrotic disorders affecting the liver and cardiovascular system (Parker et al., 2020). Given that psoriasis is an inflammatory disease, alterations in *Alistipes* could be linked to inflammatory processes impacting psoriasis development. *Lactobacillus salivarius* is distinguished by its production of bacteriocins—specifically subclasses IIa, IIb, and IId that inhibit pathogenic bacteria, also enhancing mucosal barrier function (Messaoudi et al., 2013). Species *CAG-485 sp002404675* and *CAG-83 sp000435555* have not been specifically reported in the literature. Class *Clostridia* to which they belong is believed to rebalance Th1/Th2/Th17 cells and create a less pro-inflammatory immunological milieu in the gut, leading to the accumulation of Treg cells that suppress inflammation (Chen et al., 2020). *Helicobacter* is a genus of Gram-negative bacteria. Existing literature has predominantly focused on *Helicobacter pylori*, the most well-known species commonly associated with gastrointestinal diseases. A meta-analysis revealed that the incidence of *Helicobacter pylori* infection in patients with psoriasis was 10.7% higher than that in the control group. Furthermore, psoriasis patients with *Helicobacter pylori* infection tend to have higher Psoriasis Area and Severity Index (PASI) scores. These findings indicate a significant association between *Helicobacter pylori* infection and psoriasis (Yu et al., 2019). Concerns about the relationship between *Helicobacter pylori* infection and psoriasis persist, as several studies have produced conflicting results (Halasz, 1996; Fabrizi et al., 2001; Azizzadeh et al., 2014).

Microbial taxa associated with an increased risk of psoriasis include phylum *Omnitrophota*, order *Flavobacteriales*, family *Elusimicrobiaceae*, family *Fusobacteriaceae*, family *CAG-698*, family *CAG-977* and genera such as *Bacillus AY, Brevibacillus B*, *Demequina*, and *UBA6398*, alongside specific species like *Blautia sp001304935* and *Desulfovibrio piger*. These harmful microbial components are implicated in disrupting gut barrier integrity, promoting systemic inflammation, and alternating immune regulation. In our study, several species within the family *Lachnospiraceae* were identified, including *Blautia sp001304935*, *Brevibacillus B*, *UBA3282 sp002493835*, *Blautia A sp002159835*, and *UBA7182*. *Blautia* is the main genus detected in the human intestine within *Lachnospiraceae* through metagenomic analyses (Vacca et al., 2020). Except for *UBA7182*, the others were identified as risk factors for psoriasis, which aligns with previous research reporting a decrease in *Lachnospiraceae* abundance in psoriasis patients (Chen et al., 2018; Hidalgo-Cantabrana et al., 2019). This aligns with our results (Hidalgo-Cantabrana et al., 2019; Sun et al., 2021). Family *Fusobacteriaceae* may contribute to systemic inflammation and immune dysregulation. The relative abundance of *Fusobacterium* is increased in mouse models and the colonic mucosa of Inflammatory Bowel Disease (Keku et al., 2013). The influence of *Fusobacteriaceae* on metabolic diseases can be linked to both the direct or indirect activation of inflammatory pathways and the production of bacterial metabolites (Rau et al., 2018). *Desulfovibrio piger* is linked to hydrogen sulfide production, a by-product of sulfate reduction, which can damage the gut lining and exacerbate inflammatory conditions (Rey et al., 2013).

Interestingly, we have identified for the first time a correlation between *Omnitrophica* and psoriasis. Phylum *Omnitrophica*, one of the world’s oldest and tiniest bacteria, has been poorly understood (Seymour et al., 2023). Although research on *Omnitrophica* is currently limited, this discovery is highly significant and may open new avenues for both basic and clinical research.

The presence of family *CAG-977* in bidirectional MR analyses suggests its potential importance in the field, but how it works is still unclear. Further studies clarifying the precise mechanisms through which family *CAG-977* interacts with the disease process may pave the way for more effective therapeutic and preventive strategies.

By understanding the distinct functions and modes of operation of these microbial taxa, we can explore novel therapeutic approaches for the treatment of psoriasis. Strategies including prebiotics, probiotics, or specifically targeted antibiotics, offer promising avenues for restoring microbial homeostasis, mitigating systemic inflammation, and improving the symptoms of psoriasis.

The current study possesses numerous strengths. Firstly, it effectively addresses the challenges posed by confounding factors and reverse causality through MR, thus compensating for the limitations inherent in traditional observational studies that are susceptible to such interference. Secondly, to ensure the integrity of our IVs, palindrome SNPs were excluded, maintaining the authenticity of the SNPs employed. Additionally, the study employed the F-statistic to verify the strength of these SNPs. Finally, our work utilized the latest gut microbiota database, including 473 taxa classified down to the species level, advancing the innovative aspects of our study.

Our study acknowledges several limitations. Firstly, our investigation primarily focuses on GWAS data originating from individuals of European descent. This constraint limits the ability to generalize our findings across diverse ethnic groups. Secondly, the reliability of MR analysis is heavily influenced by the validity of the chosen SNPs serving as instrumental variables. Despite rigorous selection criteria, the potential for minor instrumental bias cannot be completely eliminated, particularly given the intricate composition of the gut microbiome. Finally, the sample sizes may need to provide more statistical power to detect subtle but biologically significant associations, particularly when considering specific microbial taxa.

## 6 Conclusion

A bidirectional Mendelian randomization study has been conducted to investigate the potential causal relationships between gut microbiota and psoriasis. The results indicate that seven gut microbiota taxa exert protective effects on psoriasis, whereas twelve taxa were found to have negative causality. Additionally, reverse Mendelian randomization estimates suggest a causal link between psoriasis and 13 gut microbiota taxa. These findings highlight the need for further research to elucidate the mechanisms underlying the influence of these bacteria on psoriasis, aiming to develop targeted microbial interventions that could enhance treatment strategies and improve clinical outcomes for psoriatic patients.

## Data availability statement

The datasets presented in this study can be found in online repositories. The names of the repository/repositories and accession number(s) can be found below: the raw dataset of gut microbiota was obtained from the NHGRI-EBI GWAS Catalog (https://www.ebi.ac.uk/gwas/). The cohort of psoriasis was from FinnGen (https://www.finngen.fi/en).

## Ethics statement

Ethical approval was not required for the study involving humans in accordance with the local legislation and institutional requirements. Written informed consent to participate in this study was not required from the participants or the participants’ legal guardians/next of kin in accordance with the national legislation and the institutional requirements.

## Author contributions

MQ: Writing – review and editing, Writing – original draft. JS: Writing – original draft, Writing – review and editing. ZZ: Writing – review and editing, Validation, Methodology, Data curation, Conceptualization. DB: Writing – review and editing, Project administration, Methodology, Investigation, Formal analysis, Data curation. CT: Writing – review and editing.

## Footnotes

1
https://r10.finngen.fi/

2
https://www.ebi.ac.uk/gwas/
