# Supplementary material for: Genetic insights into the gut microbiota and risk of psoriasis: a bidirectional mendelian randomization study
Source: Front Microbiol. 2024 Aug 5;15:1434521. doi: 10.3389/fmicb.2024.1434521 (PMC11331342; doi:10.3389/fmicb.2024.1434521)
Supplement: Supplementary file 3 [file Table_3.DOCX]

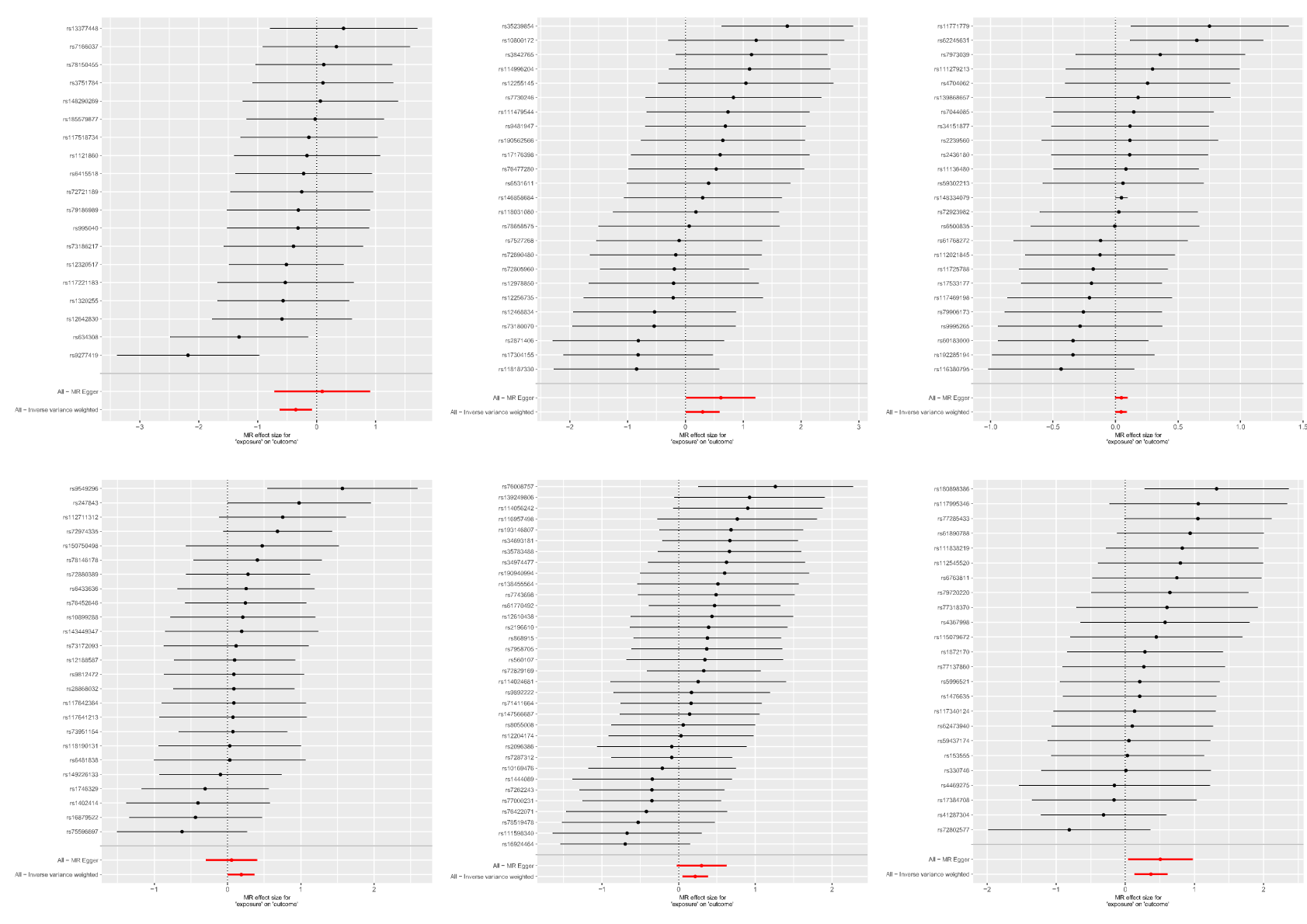


Figure S1. Forest plots for causal effects of gut microbes on psoriasis (single SNP)


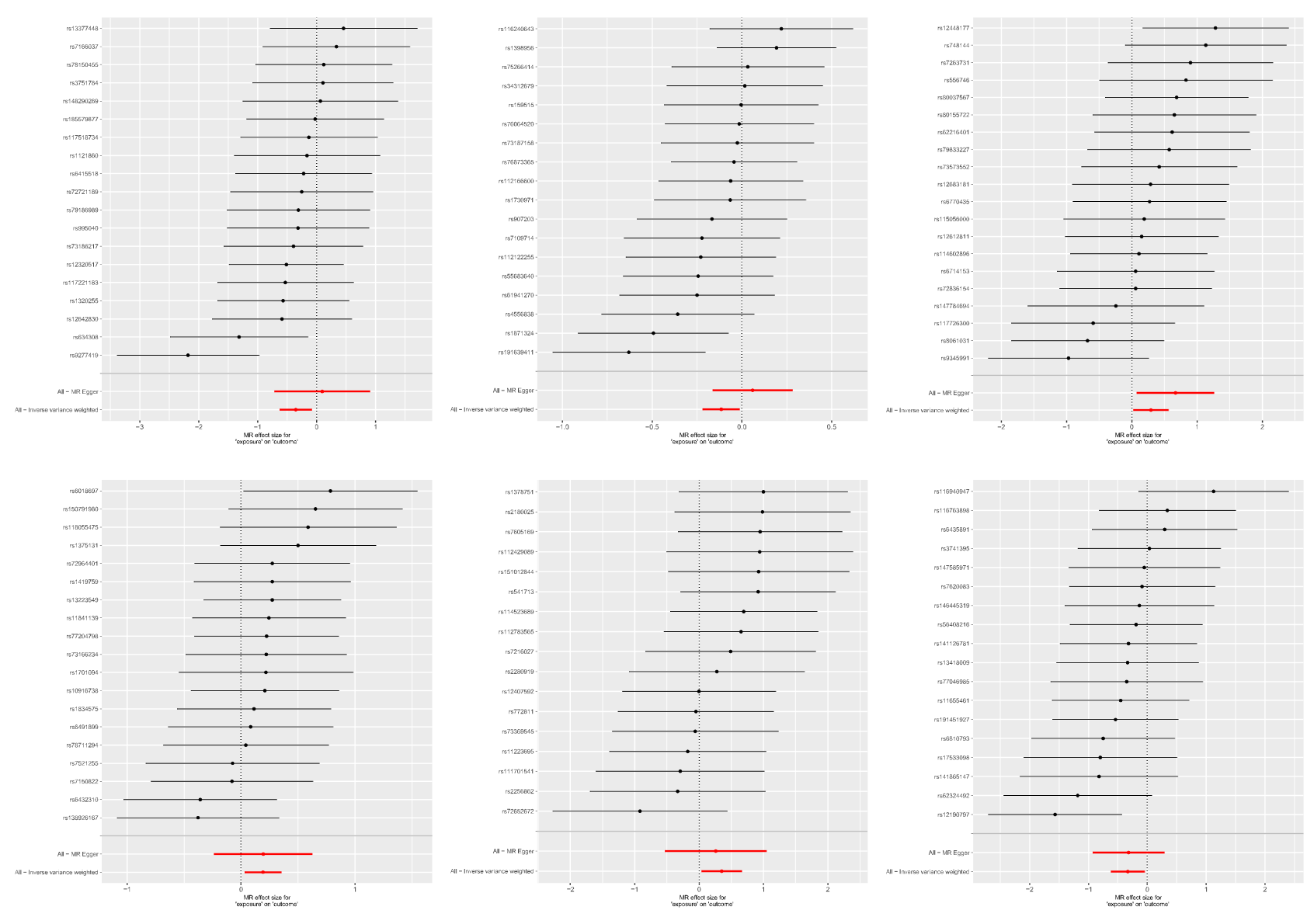


Figure S2. Forest plots for causal effects of gut microbes on psoriasis (single SNP)


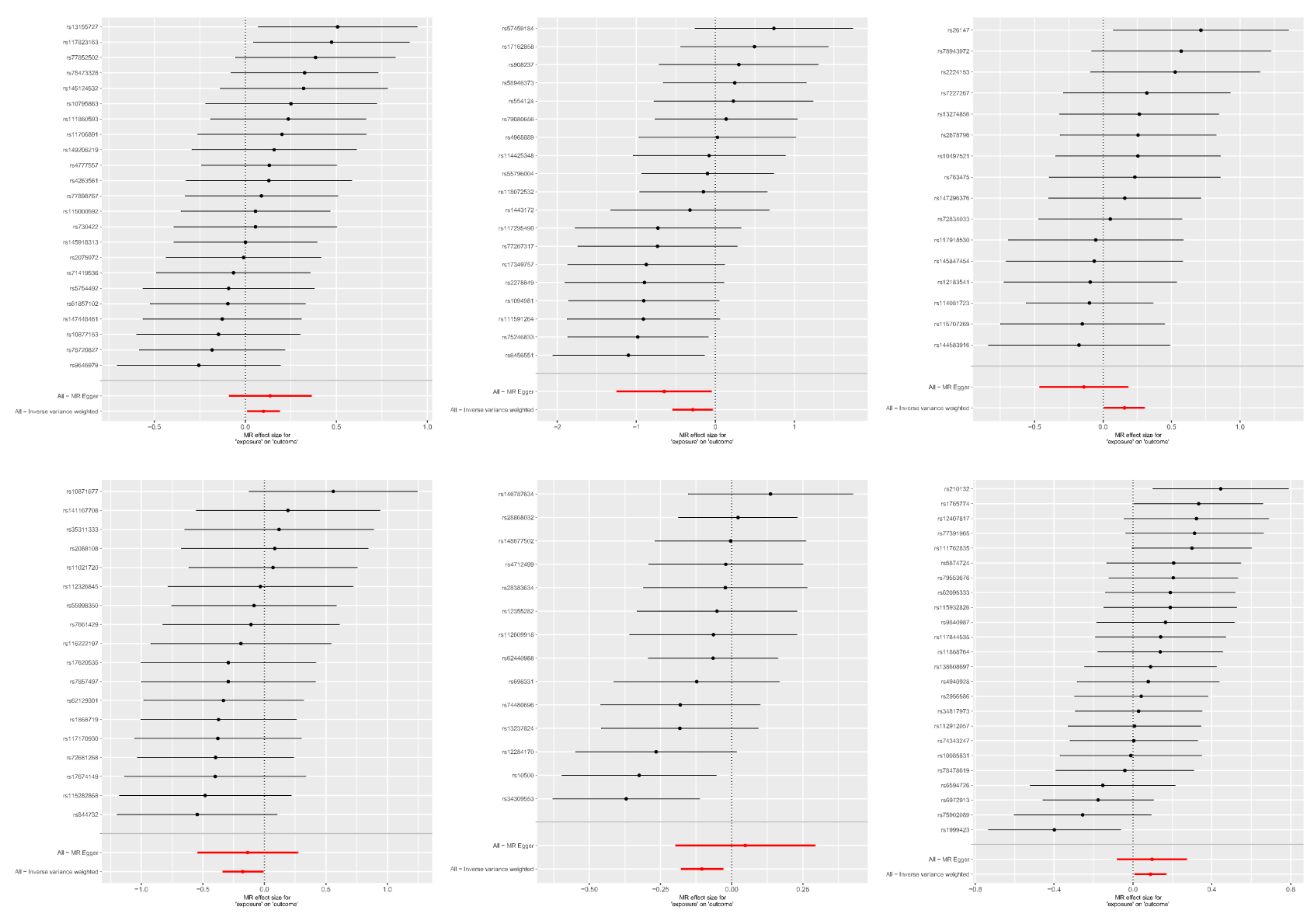


Figure S3. Forest plots for causal effects of gut microbes on psoriasis (single SNP)


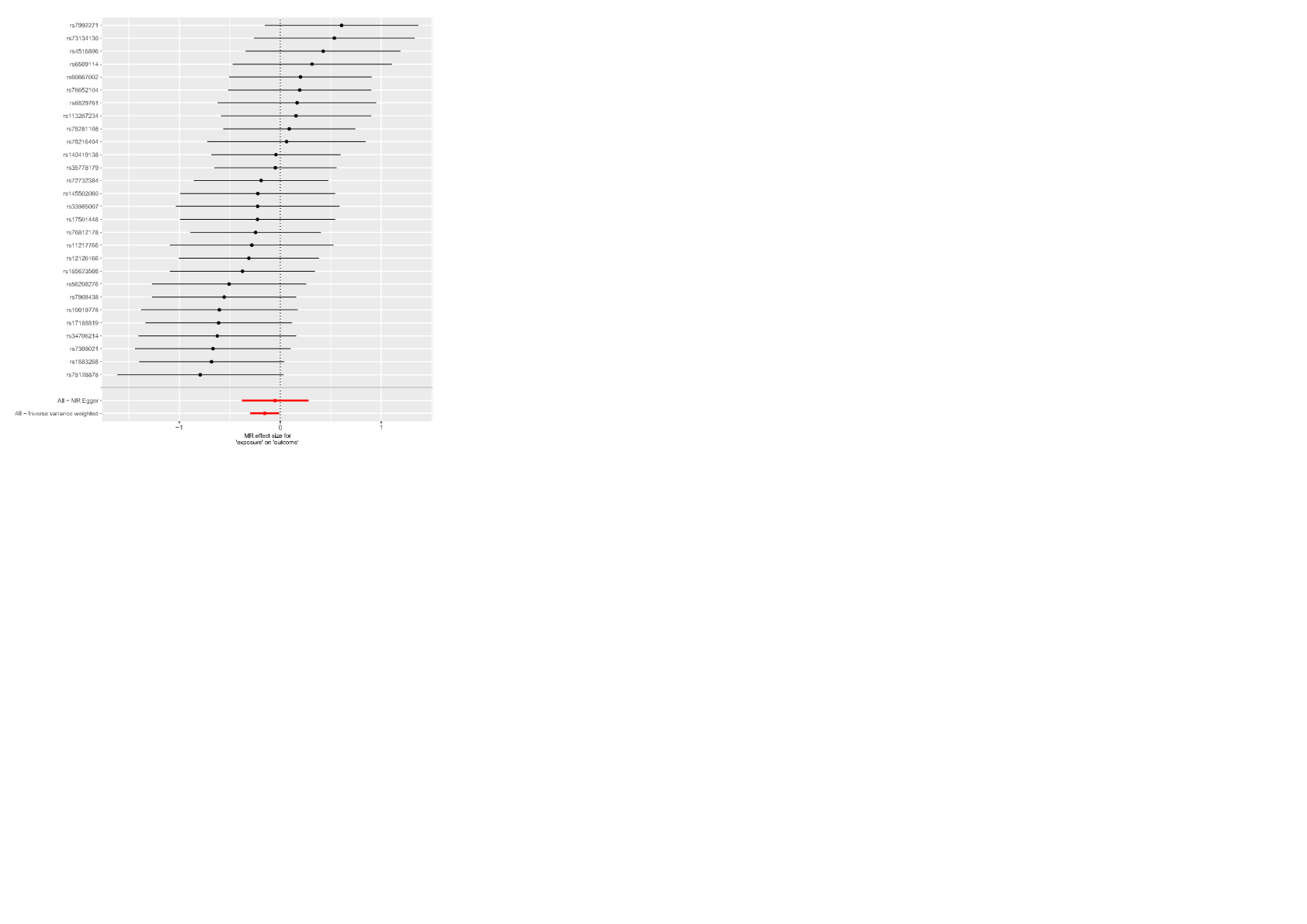


Figure S4. Forest plots for causal effects of gut microbes on psoriasis (single SNP)


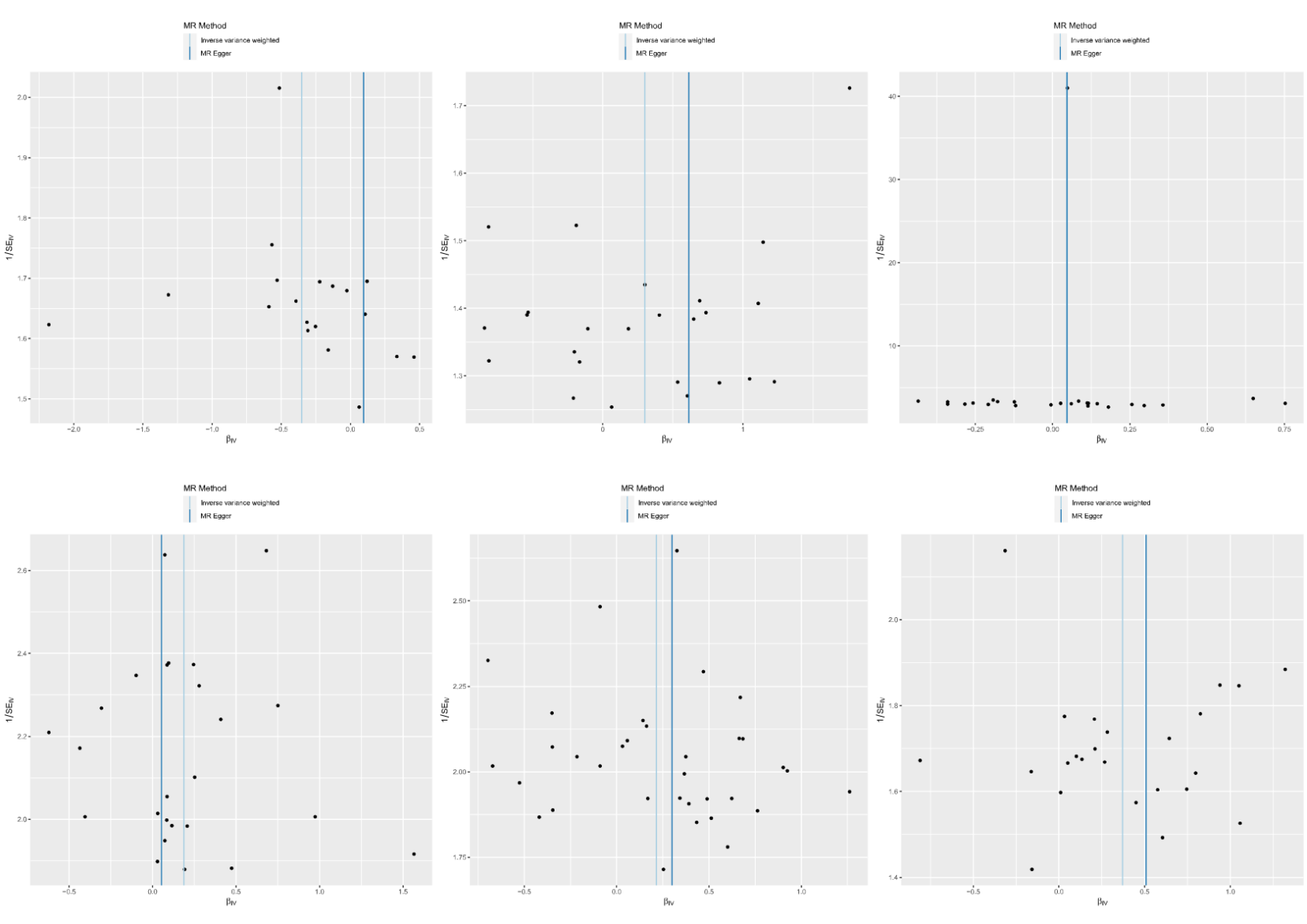


Figure S5. funnel plots for MR causal effects of gut microbes on psoriasis


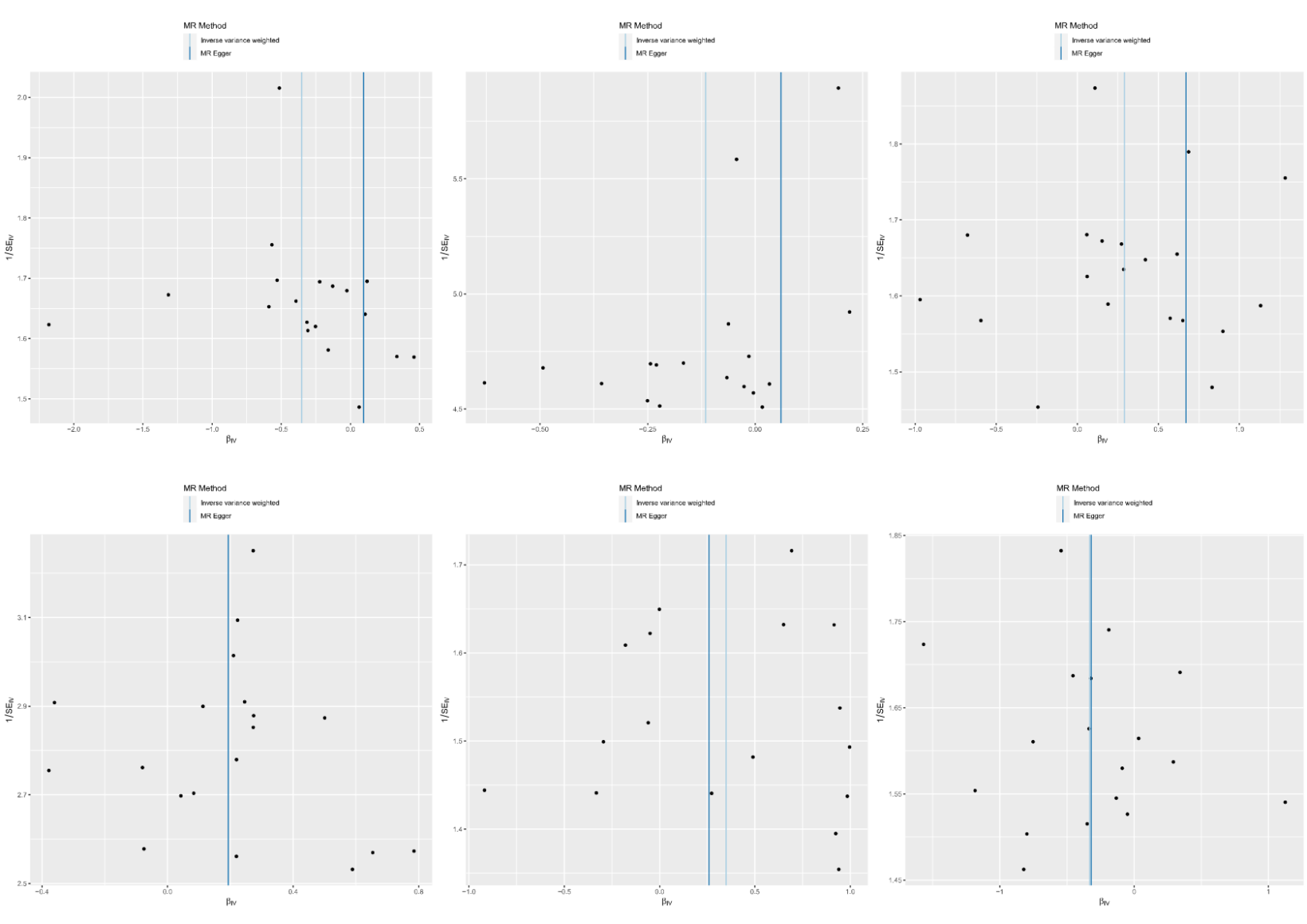


Figure S6. funnel plots for MR causal effects of gut microbes on psoriasis


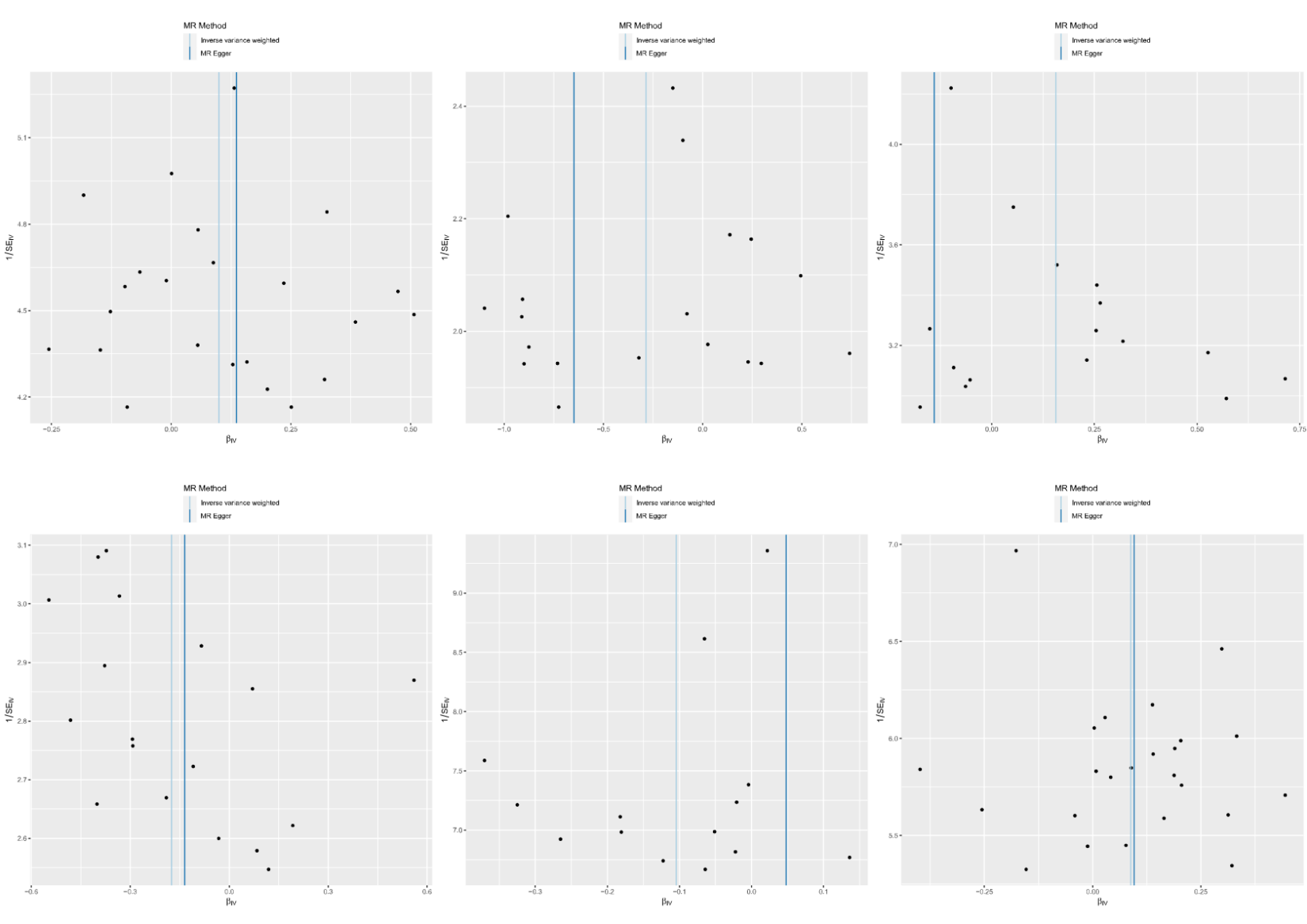


Figure S7. funnel plots for MR causal effects of gut microbes on psoriasis


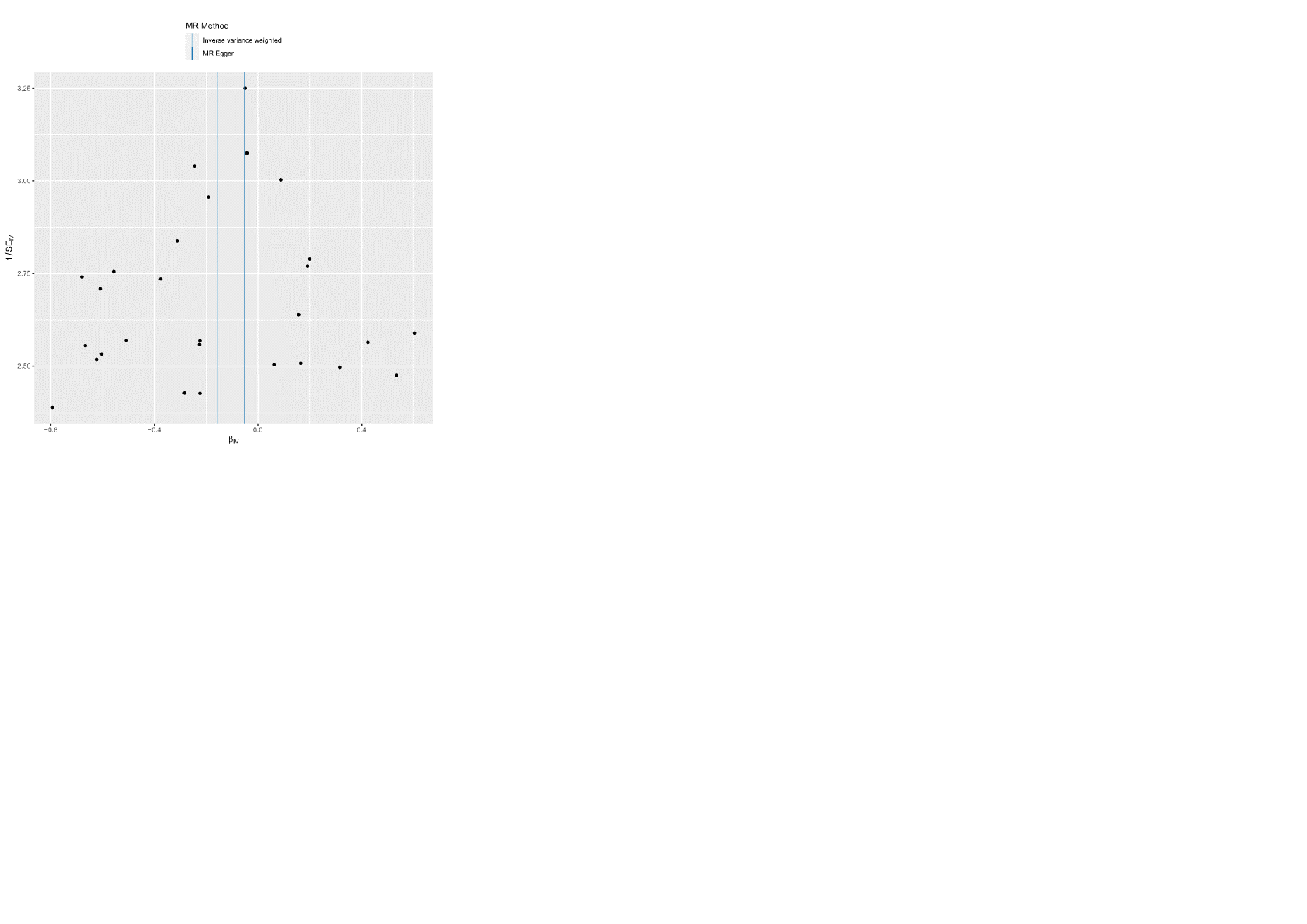


Figure S8. funnel plots for MR causal effects of gut microbes on psoriasis


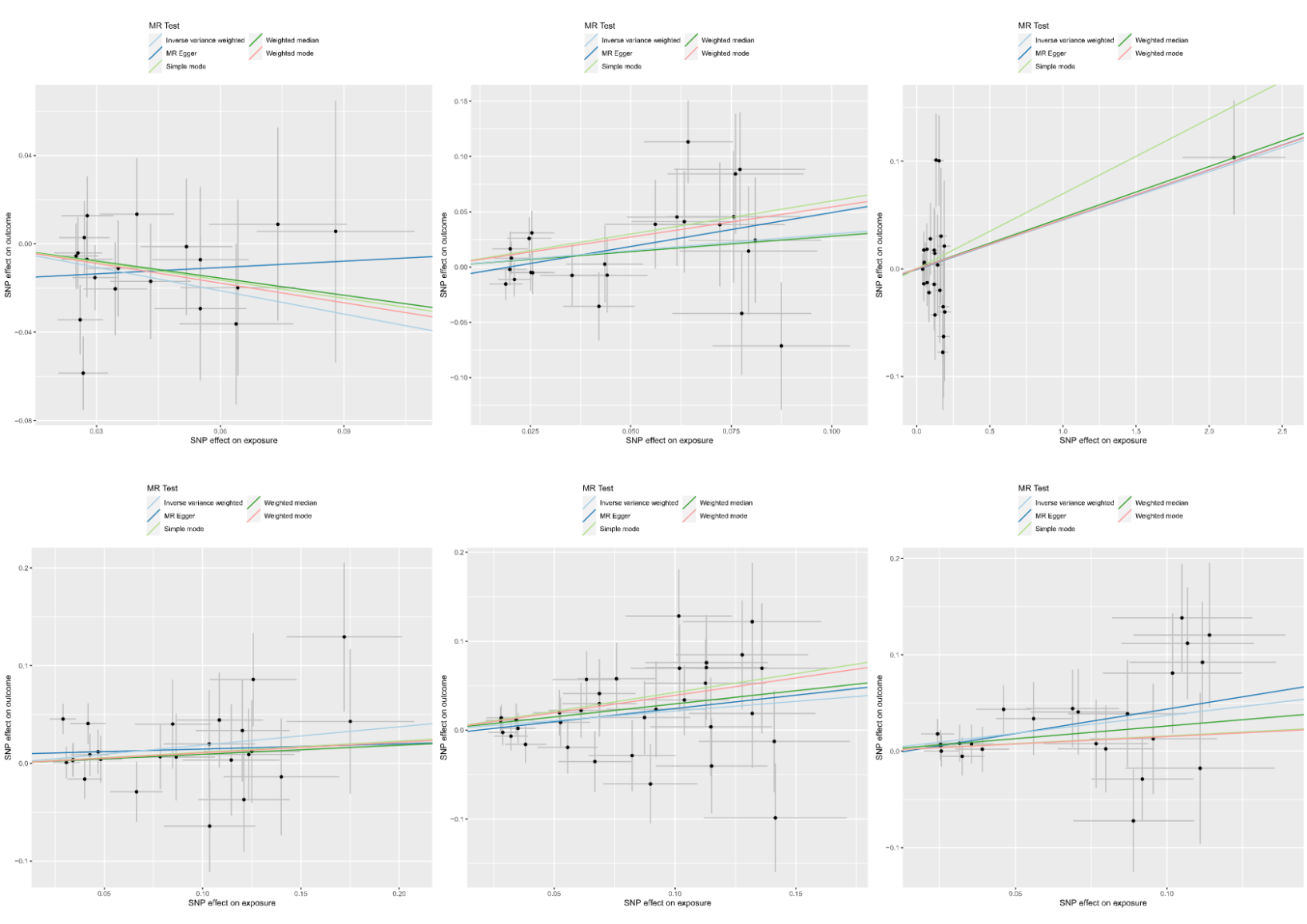


Fig S9. scatter plot visualizing MR results for genetic correlations of 19 GM taxa with psoriasis using diverse MR methods


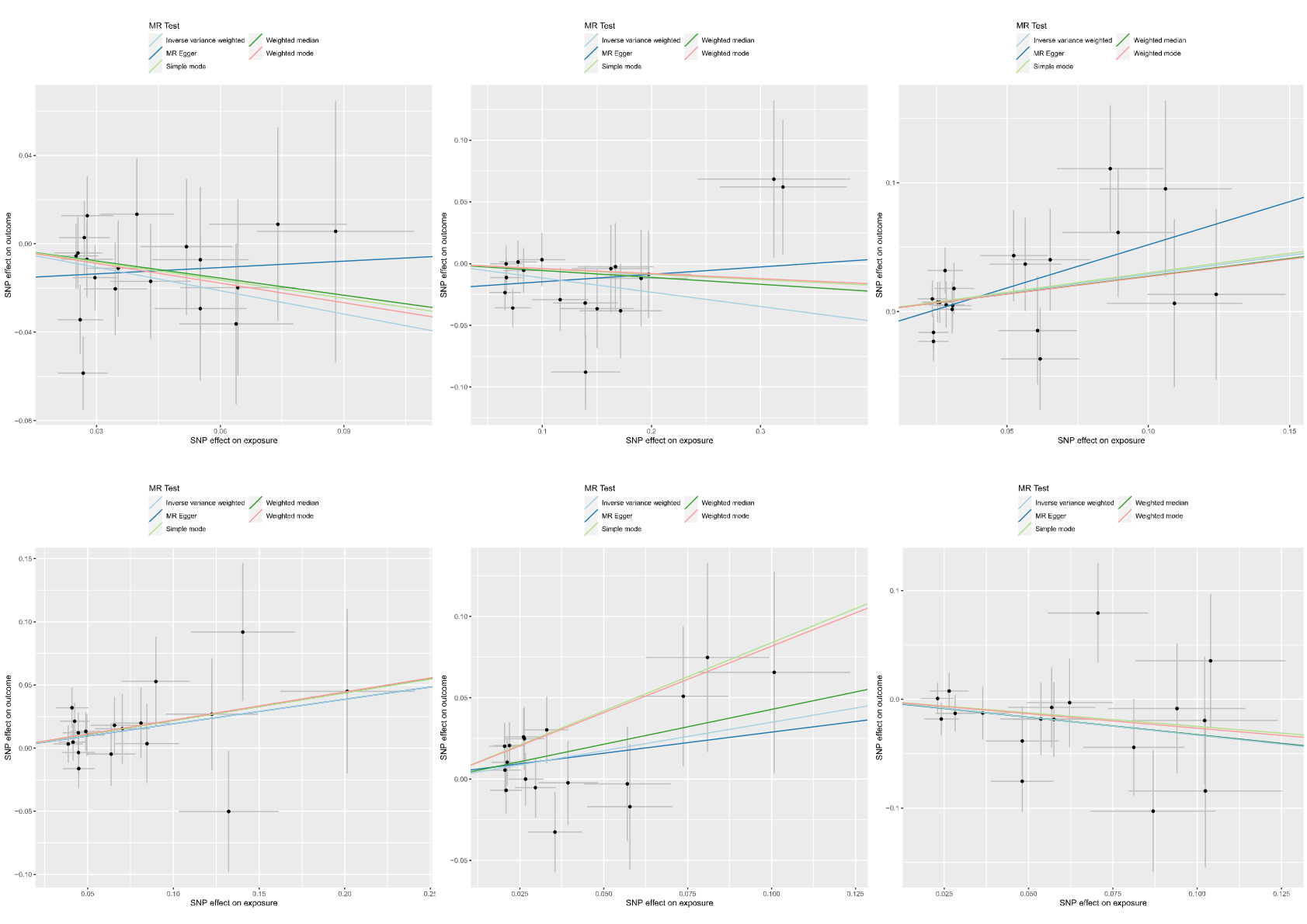


Fig S10. scatter plot visualizing MR results for genetic correlations of 19 GM taxa with psoriasis using diverse MR methods


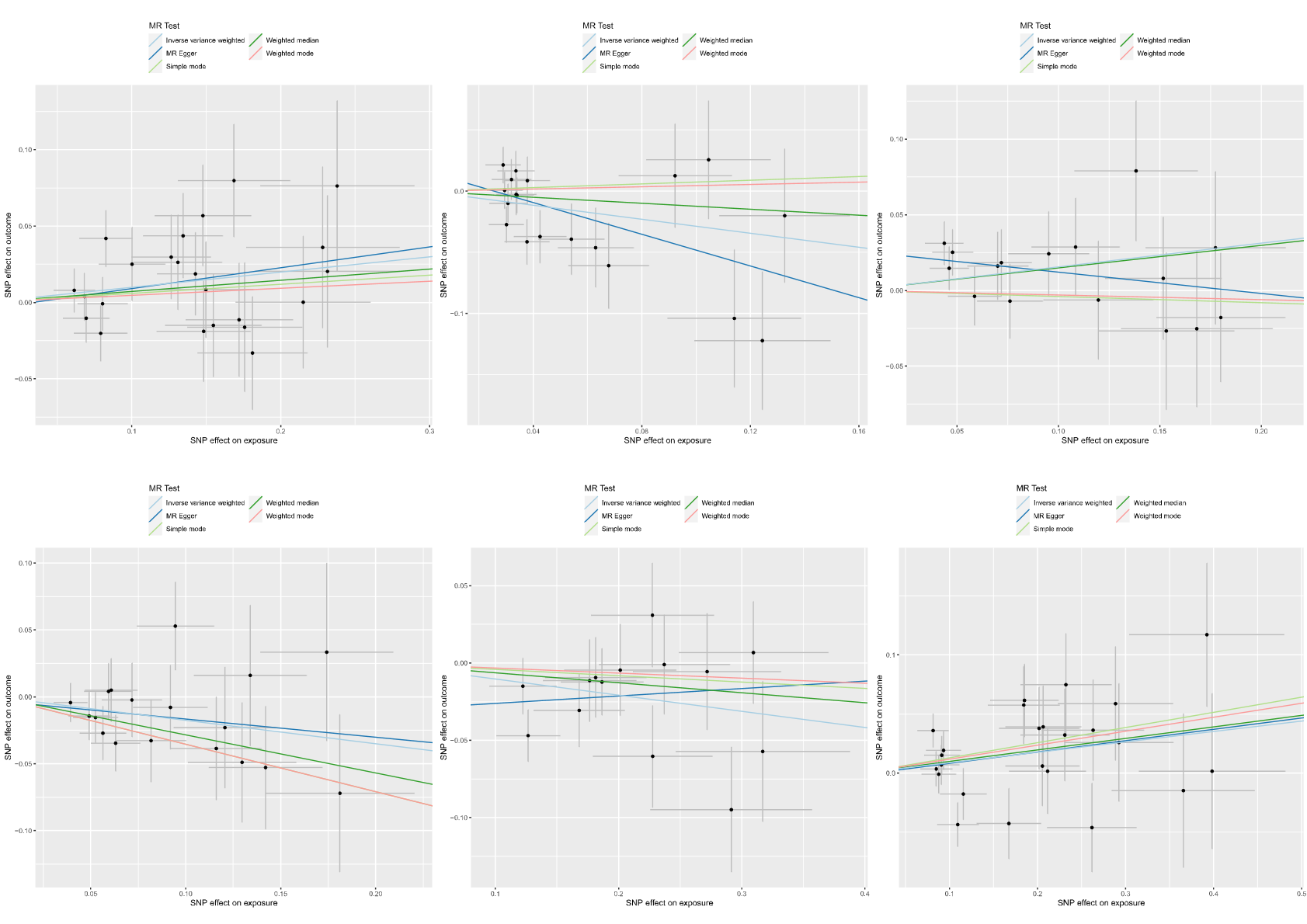


Fig S11. scatter plot visualizing MR results for genetic correlations of 19 GM taxa with psoriasis using diverse MR methods


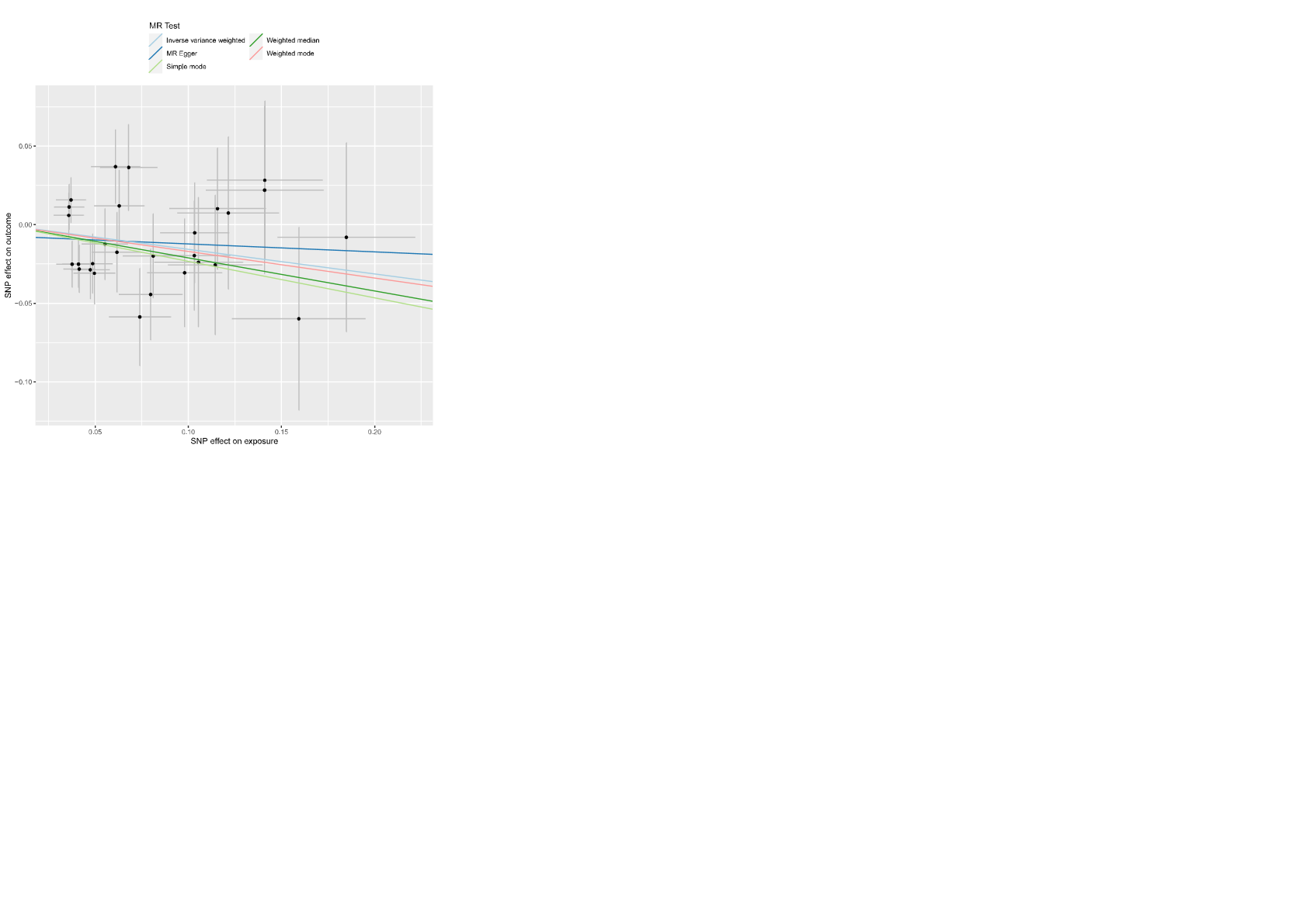


Fig S12. scatter plot visualizing MR results for genetic correlations of 19 GM taxa with psoriasis using diverse MR methods


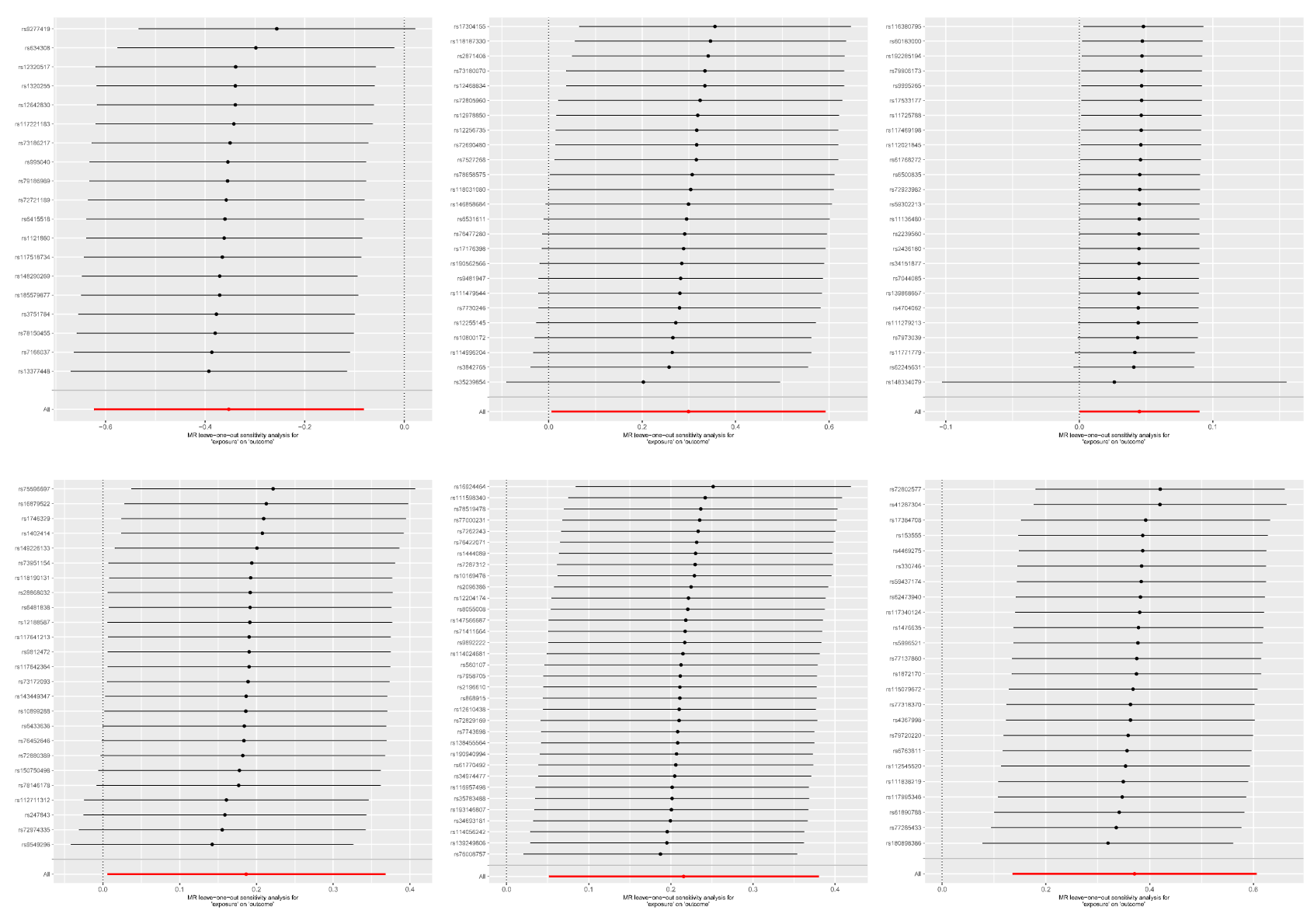


Figure S13. leave-one-out for MR causal effects of gut microbes on psoriasis


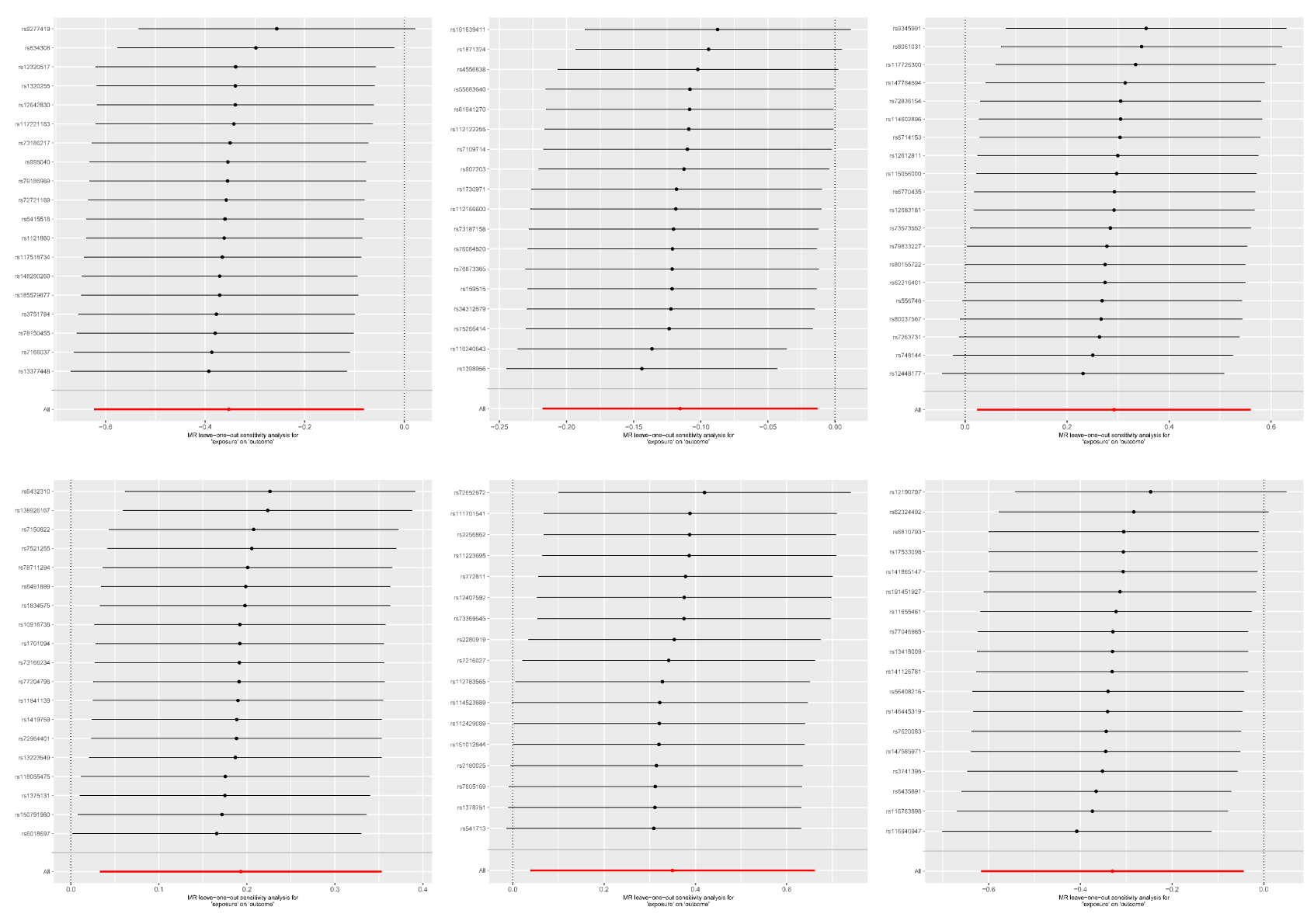


Figure S14. leave-one-out for MR causal effects of gut microbes on psoriasis


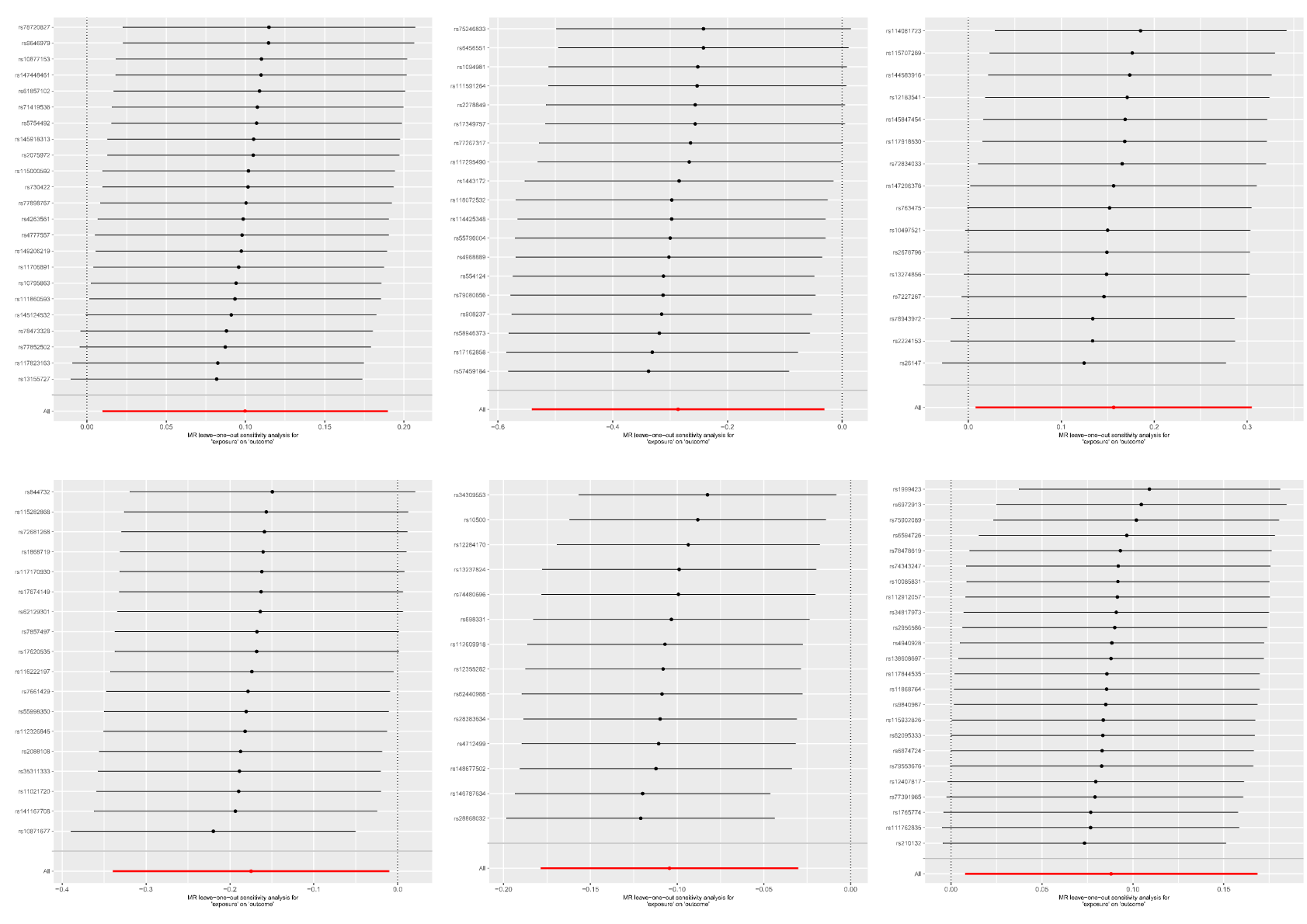


Figure S15. leave-one-out for MR causal effects of gut microbes on psoriasis


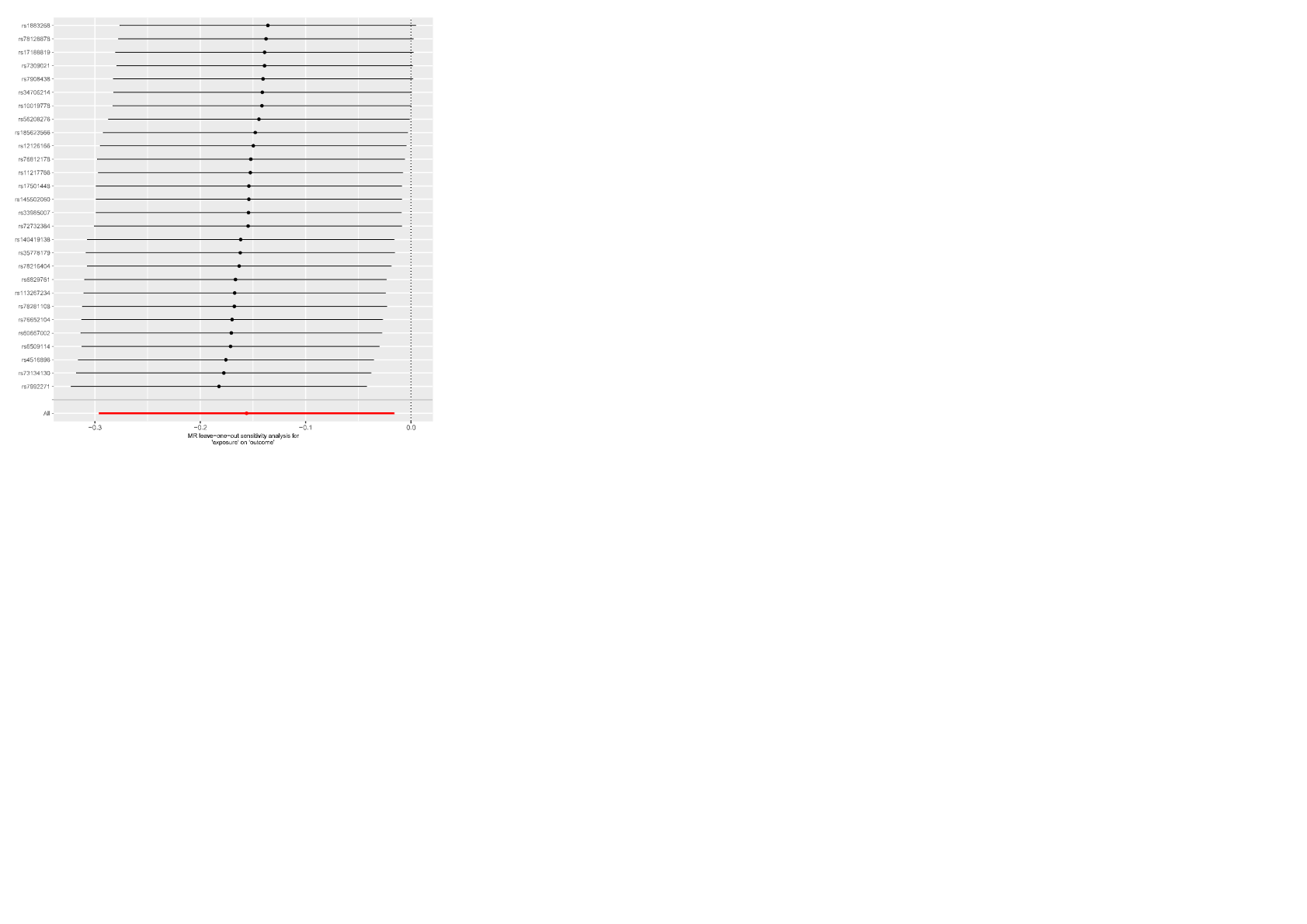


Figure S16. leave-one-out for MR causal effects of gut microbes on psoriasis
